# Empirical Study on the Impact of Government Environmental Subsidies on Environmental Performance of Heavily Polluting Enterprises Based on the Regulating Effect of Internal Control

**DOI:** 10.3390/ijerph20010098

**Published:** 2022-12-21

**Authors:** Weiqi Pei, Weiran Pei

**Affiliations:** 1 College of Economics and Management, Zhengzhou University of Light Industry, Zhengzhou 450000, China; 2 School of Marxism, Shanghai University of Finance and Economics, Shanghai 200233, China

**Keywords:** heavy-polluting enterprises, environmental subsidies, internal control, environmental performance

## Abstract

At present, China’s economy is developing rapidly; however, at the same time, it is also bringing more and more serious environmental problems. Although many laws have been established in the country to limit pollution by enterprises—and enterprises are actively saving energy and reducing pollution—the situation is still not optimistic. In 2016, there were 35 cities across the country that exceeded the annual average air quality standard; these regions have been plagued by haze for a long time, which seriously threatens people’s health and sustainable social and economic development. Therefore, while pursuing economic benefits and realizing greater value, importance must be attached to environmental performance, especially for enterprises with serious pollution. Using the panel regression analysis method, based on the data of enterprises from 2010 to 2019, this paper empirically analyzes the effects of government environmental protection subsidies and internal control quality on the environmental performance of heavily polluting enterprises, and verifies the nonlinear critical effect of government subsidies and internal control on environmental performance. In addition, this paper also uses the intermediate effect model to verify the mechanism by which environmental subsidies impact the environmental performance of heavily polluting enterprises. Through the empirical analysis, the effects of environmental protection subsidies and internal control on the environmental performance of enterprises with serious pollution are obtained. In the process of environmental subsidy affecting the environmental performance of heavy-polluting enterprises, internal control plays a key intermediary role. In addition, environmental subsidies and internal controls have a certain impact on the environmental performance of heavily polluting enterprises. Through the empirical analysis, it is concluded that there are obvious differences between government subsidies and internal control on the environmental governance effects of heavily polluting enterprises in different regions and with different property rights. Among them, the environmental protection subsidies have the greatest impact on pollution degree in the central region, followed by the eastern region; in the western region, it is not obvious. Secondly, the incentive effect of government environmental subsidies on state-owned enterprises with serious environmental pollution is better than the environmental governance effect of non-state-owned enterprises.

## 1. Introduction

President Xi Jinping, in his report to the 19th CPC National Congress on 18 October 2017 in particular, pointed out the strategic importance of China’s sustainable development. China needs to protect its economy and prevent environmental pollution and resource deterioration, which poses a huge challenge to the service capacity and level of the Chinese government [1,2,3,4,5,6]. 

Illegal environmental behavior is an important factor in China’s environmental behavior, and the most basic ethical standard for enterprises is to follow environmental regulations (ER). The institutional arrangement is an increasingly important factor next to resource allocation and technological progress. Under the new normal, China’s top priority is to “stabilize growth” [7,8,9,10]. In the absence of other drivers, China’s industrial development is particularly dependent on institutional innovation. In particular, by improving the basic economic system, we can stimulate the vitality of all forms of ownership and increase the driving force for industrial development [11]. Building a more orderly market mechanism is conducive to fair competition in the market and creates a good environment for industrial development. By improving government functions and deepening the reform of the fiscal and taxation systems, China’s industrial policy system and tax burden can be made to be more reasonable [12]. The main driver of China’s economic development is the development of urban and rural integration systems and mechanisms and the establishment of an open economic system, which exerts great pressure on China’s industrial development in addition to promoting the reform of China’s higher education, scientific research and development, the coordinated development of the secondary industry and tertiary industry, green ecological construction, and the transition of the mode of production and economic development mode [13]. The Chinese government should not only give consideration to economic and social development and environmental protection, but also coordinate the issues of energy, the environment and economic development. As far as China’s current developmental situation is concerned, the essence is the allocation of environmental resources [14]. According to the 2021 World Air Quality Report, only about 3.4% of cities around the world have air quality that meets WHO standards. The findings show that air pollution mainly comes from oil-powered transportation and coal-based industrial production, which has a serious negative impact on human health and economic development. Among the environmental problems China is currently facing, air pollution and water pollution caused by industrial production are prominent. According to incomplete statistics, 40% of the water sources in China’s seven major river systems fail to meet the quality standards for drinking water, about 30% of urban water sections have lost their functionality, and more than 70% of lake water resources are seriously eutrophicated, with serious nitrogen and phosphorus pollution which has a great inhibitory effect on economic development. In order to effectively solve the problem of environmental pollution, the Chinese government has continuously increased investment in environmental treatment and optimized subsidy policies for industrial enterprises. The total investment in environmental treatment has increased from CNY 665.42 billion in 2010 to CNY 1063.89 billion in 2020. By 2021, about 66% of Chinese cities will have significantly lower PM2.5 concentrations than in 2020, and water pollution will also be significantly improved; good results have been achieved in environmental governance. From the perspective of environmental issues, environmental deterioration has a significant inhibitory effect on economic growth, and environmental degradation should be controlled. Enlightened environmental management policies should be implemented to reduce carbon dioxide emissions and fuel consumption, and reasonable carbon emission reduction policies are very conducive to sustainable economic development [15]. From the economic point of view, China’s economic growth has slowed down, and the protection of the ecological environment is conducive to the stable development of the economy, the smooth transition of the economy and the realization of sustainable development [16]. 

Existing studies show that internal control can be engaged in two levels, prevention and post-correction, to improve environmental performance. In the case of high-quality controls, it can prevent potential managers from reducing costs and allow them to detect environmental problems in a timely manner. Therefore, determining how to establish an effective internal control system is the key to improving enterprise environmental performance. At the same time, as the external auxiliary force of enterprise environmental capital expenditure, can the environmental subsidies of the government influence the quality of internal control, so as to improve the environmental performance of enterprises? This paper aims to explore the effect of internal quality control on the relationship between government environmental subsidies and corporate environmental performance.

## 2. Research Hypothesis

### 2.1. The Impact of Internal Control Quality on Enterprise Environmental Performance

Li XI (2017) argues in the study that, although the country has intensified environmental protection efforts and increased the punishment of illegal enterprises, environmental pollution accidents are still frequent [17]. Financial aid is an important measure for the state to regulate economic and social life. With the development of society and the economy, people have higher and higher requirements for quality of life, and the awareness of environmental protection is becoming stronger and stronger [18]. The function of enterprises is not only limited to providing various services to the public, but more importantly, to play the function of environmental protection. The financial subsidies of the government are to solve the problem of pollution [19]. Under the government subsidy, enterprises will invest more funds to improve their technical level and improve their environmental performance, thus reducing the damage to the environment and promoting the sustainable development of enterprises [20]. Therefore, government subsidies can significantly improve the environmental benefits of enterprises [21]. Yan Yuehui and Song Liangrong (2020) said, in their research, market incentive environmental regulation is a market mechanism developed by the government to promote the emission of enterprises and control and optimize the overall pollution level of society [22]. The essence of market incentive environmental regulation is to set the price of pollutants emitted by enterprises, which is realized by internalizing the environmental cost [23]. To solve this problem, there are two main ways: one is to tax the environment, to avoid the ancient tax, in order to increase the production cost of enterprises, so as to achieve social and enterprise cost unification, control the purpose of pollution emission [24]. Second, in order to encourage enterprises to carry out innovation and research on clean energy technologies, the government can encourage enterprises to carry out technological innovation through high taxes or encourage enterprises to carry out technological development through subsidies [25]. From the point of view of utility, environmental tax has a certain effect in the short term, but it will also have a certain inhibition effect on pollution discharge to a certain extent [26]. Therefore, environmental subsidy, as a way of environmental control, has been widely paid attention to [27]. Li Shasha (2018) proposed, in his research, that due to environmental protection, energy conservation and emission reduction, China’s high-polluting industries have lost their price advantage in the production process, but they can still bring great benefits to the whole society in terms of environmental protection, environmental protection and other aspects [28]. As these external factors are difficult to be adjusted by the market mechanism, the government, as an economic manager, needs to intervene and correct them, and take a series of measures to promote the sustainable development of polluting industries [29]. This requires financial support in theory and a series of environmental reforms to boost the environment.

In highly polluting industries, the risks of technological innovation and green development are mainly reflected in the three aspects of investment, investment and after [30]. Technological innovation is a long process, from investment to research and development, it needs a lot of capital and manpower [31]. First, in this project, the possibility of future research results is uncertain; Secondly, whether the scientific research results can be recognized by the industry, the development of the industry is a long process, often not a one-time investment can be achieved; if the results of research and development cannot reach the development of the industry, then the initial investment will cause huge losses [32]. Finally, even if this research result is recognized by the industry, whether it will change the supply and demand pattern in the future and whether it will change economic policies will have a certain impact on the company’s profit and market value in the future [33]. Therefore, the following hypothesis is proposed:

**Hypothesis** **1:** *Government environmental subsidies promote the improvement of enterprise environmental performance.*

### 2.2. The Impact of Government Environmental Subsidies on Corporate Environmental Performance

Li Chenying (2019) proposed, in his study, that the country has a strengthened awareness of environmental protection at the macro level, while at the micro level, enterprises are the biggest source of pollution. As a result, both governments and citizens are placing higher expectations on businesses, enterprises are expected to reduce environmental pollution through green production, green research and development, recycling and other ways [34]. Sustainable development and more stringent environmental protection policies are to strengthen the understanding of the environment and strengthen environmental management. Environmental governance and green development will inevitably increase the cost of enterprises directly or indirectly, which will have a certain impact on the short-term business performance of enterprises [35]. In theory, the most fundamental purpose of enterprise governance is to ensure the reasonable operation and management of enterprises, while the highest goal of enterprises is to promote the development of enterprises. For serious polluting enterprises, the environmental protection department has a lot of relevant laws and regulations, if the enterprise violates the regulations, it is the enterprise’s internal control that is not in place [36]. In order to achieve the basic purpose of the enterprise, the relevant laws and regulations must be strictly followed. With increasingly strict environmental regulations and increasing public awareness of environmental protection, enterprises should take environmental protection measures to prevent environmental risks [37]. According to Liu Donghong (2021), General Secretary Xi Jinping, times stressed that “clean water and lush mountains are gold and silver mountains” is the basic principle of development, but the illegal emission of enterprises violated this concept, damaging the rights and interests of the public [38]. In enterprises, there is a serious conflict between environmental protection and economic interests, and with the increase of government control, the economic losses are also increasing, which has caused a non-negligible negative impact on the development of enterprises [39]. In this new situation, enterprises must abandon the previous management mode of “government punishment, enterprises deal with after the event”, and actively implement environmental management strategies [40]. However, the management of many enterprises feel that they have invested a lot of money and manpower in environmental management, and there are still some doubts about whether environmental governance can be improved [41]. Through this series of studies, not only make the enterprise operators realize that the practice of environmental management can improve the environmental performance of the enterprise, but also can promote the innovation ability of the enterprise, reduce the cost, and give play to the rationality of the organization [42]. Zheng Jia (2021) also said in the study that the specific practice of enterprises to implement environmental responsibility is their environmental capital expenditure [43]. In the investment of environmental protection, enterprises often spend a huge amount of money to provide a huge amount of funds for environmental protection facilities and technological innovation. This is a non-economic, long-term investment that is hard to come by in the short term [44]. 

According to neoclassicism, a corporation is a for-profit social organization whose purpose is to obtain the maximum profit [45]. In the absence of institutional and fiscal incentives, companies often have little incentive to invest in environmental protection [46]. Therefore, in order to promote the initiative of enterprises to take environmental responsibility, we must have adequate financing channels. "Enterprise Internal Control Supporting Guidelines No. 4—Social responsibility”, points out the huge losses caused by insufficient investment in environmental protection and overuse, and puts forward the establishment of the environmental protection assessment system and other specific requirements, in order to promote enterprises to assume environmental responsibility [47]. This standard can not only regulate the decision-making behavior of enterprises and strengthen the organizational structure of enterprises, but also enable enterprises to combine with the needs of various stakeholders and corresponding environmental responsibilities [48]. Therefore, the following hypothesis is proposed:

**Hypothesis** **2:** *There is a positive correlation between environmental performance and internal control quality.*

### 2.3. The Relationship between Internal Control Quality and Government Environmental Subsidies and Firm Environmental Performance

According to the research results of Yiliqi, Li Tao and Zhang Ting (2020), at the internal level, enterprises should make full use of their advantages of scale, reduce the cost of environmental protection, make full use of the advantages of capital, technology and human resources, and promote the green transformation of enterprises, therefore, it is necessary to strengthen the internal control of enterprises and enhance their awareness of environmental protection [49]. In terms of external policies, the government should strengthen the supervision and regulation of environmental protection. We should increase support for small- and medium-sized enterprises and create a favorable policy environment for their development [50]. Environmental subsidies are an important way for enterprises to obtain funds. The quality of their internal control often has a direct relationship with their environmental performance. The environmental management level of enterprises is closely related to its own level [51]. To this end, it is necessary to conduct in-depth research on internal control, environmental management capacity and environmental performance. Once the media discloses the environmental pollution problem of the enterprise, it will not only damage the image of the enterprise, but also have a negative impact on the investors who are waiting on the sidelines. In addition, it will lead to the decline of the stock price of the enterprise, thus damaging the rights and interests of the existing shareholders [52]. Wang Xingfen (2020) proposed, in his study, that environmental regulation, market structure and management cognition would all have a certain impact on environmental behavior [53]. The first is external. In order to reduce the cost of environmental violations, the company will invest some money in environmental protection. Among them, there is a U-shaped relationship between the intensity of environmental control and the environmental input of the company. In other words, the environmental control of the local government has a “threshold effect” on the environmental input of the company [54]. 

Nowadays, with the continuous emergence of green consumption and green production concepts, some enterprises spontaneously invest in environmental protection in order to build their own green image, and their environmental protection investment is also closely linked with the national environmental protection policy. The green consumption concept of consumers will have a certain impact on the environmental investment of the company, while the environmental protection of competitors will have a positive promoting effect [55]. Second, there are internal reasons. It is mainly divided into performance evaluation index system, financial ability, debt ratio and so on. It is argued that bigger companies and better financial conditions could promote their green practices, which would not only increase their costs but also help them become more competitive [56]. The financial status of an enterprise is directly related to whether it actively implements environmental behavior. Its motivation is profit maximization and reputation, while profitability will have an impact on its environmental investment behavior, and good economic performance will encourage manufacturing enterprises to improve the environment [57]. Sun Zao and Qu Wenbo (2019) said in their research that economic transformation and technological innovation should play a positive role in energy conservation and emission reduction when formulating environmental regulation policies and measures from the perspective of “green” industrial structure and technological innovation [58]. It is found that environmental regulation forces technological innovation to have an obvious energy-saving effect, but environmental regulation cannot effectively promote the economic transformation of enterprises. Therefore, in the formulation and application of environmental regulation policies, we should give full play to the energy-saving potential of the industry, improve the production technology, especially the innovation of green technology, and promote the optimization of the product structure and structure of enterprises [59]. Through the government’s environmental control measures, we will establish a long-term mechanism for enterprise energy conservation and emission reduction, and accelerate industrial restructuring. In terms of quality, it mainly starts from the enterprise’s own energy-saving potential, and uses technology to save energy to reduce energy consumption in key energy industries, so as to improve the energy utilization rate of the whole industry [60]. In terms of total volume, we should adjust the proportion of various industries, reduce the number of industries with high energy consumption, and vigorously develop low-energy, recycling and high-tech industries. In the context of global economic integration, we should focus on cultivating competitive industrial clusters to promote energy efficiency, energy conservation and emission reduction, and realize the optimization of domestic industrial structure and industrial upgrading. Therefore, the following hypothesis is proposed:

**Hypothesis** **3:** *The quality of internal control plays a moderating role between government environmental subsidies and corporate environmental performance.*

## 3. Research Design

### 3.1. Chinese Enterprise Data Source

This paper takes a-share enterprises in Shanghai and Shenzhen stock markets from 2010 to 2019 as the research object (see Table 1), and takes the data service platforms of G Tai’an Securities and China Securities Academy as samples. This is because the region is an important link between the 11th and 12th Five-Year Plans. This year is a big year for environmental planning [61,62,63]. In order to ensure representative data, the following points were screened:

(1) Sample enterprises marked S, ST, *ST, SST, S*ST are excluded to prevent abnormal values;
(2) Excluding financial, insurance and comprehensive listed enterprises;
(3) Screening of listed enterprises lacking relevant information;
(4) On this basis, the relevant variables were shortened by 1% to avoid the influence of extreme values.

Through the screening and sorting of the above data, a total of 4080 valid samples from 2010 to 2019 were obtained.

### 3.2. Selection of Variables

In addition to ego and regulation, there are other factors in the environmental performance of enterprises. In this paper, the company’s age, the growth rate of operating income, debt level, return on total assets, cash holding level, operating efficiency, nature of equity, he executive compensation, proportion of independent directors, senior management change, and two concurrent positions are selected. Dummy variables for YEAR (YEAR) and industry (IND) are introduced. Table 2 lists the definitions and descriptions of the variables.

### 3.3. Model Design

This research model is based on the correlation between i and t, through the establishment of a linear regression equation based on i and t to forecast the method. Market phenomena are often affected by multiple factors rather than a single factor. Therefore, the use of the unitary linear regression forecasting method needs to comprehensively consider the impact of various factors on market phenomena. Only when one of the many influencing factors has a significantly greater impact on the dependent variable than other factors, the unitary linear regression method can be used for market prediction.

The prediction model of unitary linear regression analysis is:

$$Y_{i}=ax_{i}+b$$

where $x_{i}$ represents the value of the independent variable in period i; $Y_{i}$ represents the value of the dependent variable in period i; a and b represent the parameters of the unitary linear regression equation.

Parameters *a* and *b* can be obtained by the following formula:

$$b=\frac{\sum Y_{i}}{n}-a\frac{\sum x_{i}}{n}$$

$$a=\frac{n\sum x_{i}Y_{i}-\sum x_{i}\sum Y_{i}}{n\sum x_{i}^{2}-{\left(\sum x_{i}\right)}^{2}}$$

What $Y_{i}$ and $x_{i}$ mean: $x_{i}$ increases by an average of one unit, $Y_{i}$ increases by an average of a unit.

Two or more independent variables are called multiple linear regression. Since the units of each independent variable are different, the factor before the independent variable does not indicate the importance of the factor. Firstly, all variables, including dependent variables, are converted into standard values, and then linear regression is carried out. The regression coefficient obtained can reflect the importance of the corresponding independent variables. At this time, the regression equation is called the standard regression equation, and the regression coefficient is called the standard regression coefficient, which can be expressed as follows:

$$Z_{y}=\beta_{1}Z•1+\beta_{2}Z•2+\cdots +\beta_{k}Z•k$$

In this paper, the rationality and validity of the method are verified by referring to the relevant literature and the test method of theoretical results. Based on this, the model needed for the demonstration is designed.

Based on the correlation analysis between government environmental subsidies and corporate environmental performance, this paper draws the following model (1):

(1) $$CEP_{i,t}=\alpha_{0}+\alpha_{1}ESUB_{i,t}+\alpha_{i}\Sigma CONTROLS_{i,t}+\Sigma YAER_{j}+\Sigma IND_{\tau }+\varepsilon_{i,t}$$

In this case, the bottom label I represents the ith company, and the bottom label T represents the *t* period, $\varepsilon_{i,t}$ is a random error. $\alpha_{1}$ represents the relationship between government subsidies for environmental protection and corporate environmental performance. If $\alpha_{1}$ > 0, this shows that the government’s environmental protection subsidy can effectively improve the environmental performance of enterprises. If $\alpha_{1}$ < 0, it shows that the government’s environmental protection subsidy has a certain inhibitory effect on the company’s environmental performance.

When all factors $\alpha_{1}$ before the quality of internal control are positive, it indicates that the higher the company’s environmental performance, the higher its internal control quality; on the contrary, the company’s environmental performance has a significant negative correlation with the quality of the company’s internal control.

In order to test the relationship between internal control quality and enterprise environmental performance, the following model (2) is established on this basis:

(2) $$CEP_{i,t}=\alpha_{0}+\alpha_{1}IC_{i,t}+\alpha_{i}\Sigma CONTROLS_{i,t}+\Sigma YAER_{j}+\Sigma IND_{\tau }+\varepsilon_{i,t}$$

Using the moderating effect theory, the moderating factor IC and the cross-factor ESUB* IC were introduced into the relationship between government environmental subsidies and internal control quality, and the following model (3) was obtained:

$$\begin{array}{l} CEP_{i,t}=\alpha_{0}+\alpha_{1}ESUB_{i,t}+\alpha_{3}ESUB_{i,t}*IC_{i,t}\\ +\alpha_{i}\Sigma CONTROLS_{i,t}+\Sigma YAER_{j}+\Sigma IND_{\tau }+\varepsilon_{i,t} \end{array}$$

Among them, the ESUB* IC regression coefficient $\alpha_{3}$ of the transportation project reflects the influence of the government’s environmental subsidy and internal control quality on the company’s environmental performance. The author expect $\alpha_{3}$ to be significantly positive. This paper believes that there is a significant positive correlation between the company’s environmental subsidy and the company’s environmental performance.

## 4. The Empirical Analysis

### 4.1. Correlation Analysis

Table 3 shows the correlation tables of government environmental subsidies, internal control quality and enterprise environmental performance. Correlation analysis is used to investigate the collinearity between multiple variables, as can be seen from the above table, the correlation coefficients among all variables are very small, and most of them are below 0.5. Therefore, it can be preliminarily determined that the multicollinearity among all variables is very small [64,65,66,67]. As shown in Table 3, there is a 0.145 correlation between government environmental subsidy (ESUB) and enterprise environmental performance (CEP), indicating that there is a positive correlation between government environmental subsidy and enterprise environmental performance. The larger the amount of government environmental subsidy, the better the enterprise’s environmental performance, this is consistent with the author’s assumption. At the same time, the correlation between the quality of internal control and the environmental performance of enterprises also reached 0.129, which proved that the environmental performance of enterprises has a great relationship with the quality of internal control.

### 4.2. Regression Test of Regional Differences between East and West China

In order to explore the differences in the moderating effects of government environmental subsidies on the environmental performance and internal control quality of enterprises under the conditions of regional differences, the empirical sample is divided into the eastern part and the central and western part, and the regression analysis is conducted [68,69,70]. Table 4 shows the specific regression results.

First of all, Table 4 shows that in model (1) in eastern China, the relationship between enterprise environmental performance and government environmental subsidies is 0.021. In the Midwest, corporate and government subsidies for environmental protection is only 0.031 [71,72,73,74,75]. Thus, in the Midwest regions of China, the government environmental subsidy has a great role in promoting environmental benefits to enterprises. Secondly, model (2) shows that there is a significant positive effect in both the East and the Midwest. Finally, model (3) finds that in the eastern region, the regression coefficient of the environmental performance of enterprises and the intersection term ESUB* IC is 0.004, and its statistical value is 5%. In the central and western regions, it was 0.010 and statistically significant at the 1% level. This indicates that the quality control effect of internal control of enterprises in the central and western regions is more obvious, so assumption 2 has not been confirmed [76,77,78,79,80]. This may be because the eastern enterprises have more advantages than the central and western enterprises in terms of resource endowment. However, due to the huge funds and environmental protection subsidies, this will lead to the work pressure and vigilance of managers, resulting in blind investment, and even abuse of power and fraud [81,82,83,84,85]. On the other hand, due to a chronic shortage of Midwestern United States Midwest companies, environmental subsidies can reduce the burden of enterprises in environmental protection, and at the same time, more funds can be obtained in the future. In this way, the internal governance of enterprises in the central and western regions can be effectively promoted, and investment in the environment can be increased, so as to improve their environmental benefits.

### 4.3. Industrial Difference Regression Analysis

The existence of different industries represents the existence of different corporate business philosophy and commitment to environmental protection. Heavy-pollution industry is a kind of high energy consumption, high-pollution industry, and fulfilling environmental responsibility is the key to improve environmental quality [86,87,88,89,90]. In order to study the impact of different industry types on government environmental subsidies and enterprise environmental performance, this paper divides the empirical samples into two types, conducts multiple regression analysis, and obtains different results. Table 5 shows the specific regression results.

First of all, Table 5 shows that in mode (1), the government subsidy coefficients of heavy-polluting enterprises and non-heavy-polluting enterprises show a positive correlation, indicating that the state subsidy has a significant improvement effect on both heavy-polluting enterprises and non-heavy-polluting enterprises, and the environmental protection subsidy of non-heavy-polluting enterprises has a better environmental protection effect than that of polluting enterprises, indicating that the direct impact of government environmental protection subsidies on non-heavy-polluting enterprises is more obvious [91,92,93,94,95,96,97]. Secondly, model (2) shows that the internal control quality of highly polluting enterprises has a greater impact on environmental performance, while in less-serious-polluting enterprises, its impact factor is positive but not obvious, the reason is that non-heavy-polluting enterprises have better environmental performance, and the promotion effect of internal control is weaker than that of heavy-polluting enterprises. Finally, it can be seen from model (3), that the regression coefficient between the environmental performance of polluting enterprises and the intersection term ESUB* IC is 0.008, and the statistical level is 1%. However, there is a positive correlation between the environmental performance of non-serious polluting enterprises and ESUB* IC, but the level is not significant. This indicates that the regulatory effect on internal quality is more significant in a severely polluted environment, and hypothesis 3 is verified.

### 4.4. Property Rights Differential Regression Test

Different enterprises have different property rights, different business strategies and directions, and their acceptance of risks will be different. In order to explore the differences in the regulatory effects of government environmental subsidies on the environmental performance and internal control quality of companies in the case of property rights differences, the author divided the empirical samples into state-owned enterprises and non-state-owned enterprises, and conducted multiple regression analysis, respectively.

First, Table 6 shows that: Model (1), environmental subsidies of both enterprises and non-state-owned enterprises in China, have a significant promoting effect on environmental performance; Secondly, model (2) shows that the quality of internal control in both soes and non-soes has a significant positive impact on the environmental performance of the company. There were significant effects at both the 1% and 5% statistical levels. Finally, in model (3), the regression coefficient between the environmental performance of non-state-owned enterprises and ESUB* IC is 0.023, while the regression coefficient between environmental performance of state-owned enterprises and ESUB* IC is 0.015.This indicates that the quality control effect of internal control is more obvious in the governance structure of non-state-owned enterprises. The reason is that there is no natural “blood relationship” between non-state-owned enterprises and the government. Therefore, in order to maintain the relationship with the government, it is necessary to provide the necessary resources to the government and maintain a good relationship with the government. At the same time, private enterprises will seize the opportunity to increase investment in environmental protection in order to achieve sustainable development.

## 5. Conclusions and Recommendations

### 5.1. Conclusions

Based on the resource-based theory, externality theory, stakeholder theory, corporate social responsibility theory and other theories, this paper conducts an empirical analysis of Chinese-listed companies from 2010 to 2019. Secondly, the influence mechanism and methods of government environmental protection subsidies on environmental performance are analyzed: The relationship between government environmental subsidy and environmental performance was studied based on the quality of internal control. The main conclusions of this paper are:

(1) Government environmental subsidies play a good role in promoting the environmental performance of both heavy-polluting and non-heavy-polluting enterprises. Compared with heavy-polluting enterprises, government environmental subsidies have a more obvious impact on non-heavy-polluting enterprises.

(2) In ESUB* IC, the cross coefficient between the quality of internal control and the government environmental subsidies is positive, indicating that the quality of internal control as the regulatory variable strengthens the influence of the government environmental subsidies on the environmental performance of enterprises. In the heavily polluting enterprises, the moderating effect of the quality of internal control is more obvious, although the non-heavily polluting enterprises also have a positive effect, but the results were not remarkable.

(3) Regional location, the industry type, property right nature and other factors play a role to some extent in environmental subsidies, environmental performance, internal control of enterprises and other aspects of regulation. Compared with enterprises in the eastern region, government environmental subsidies in the western region have a greater positive impact on the environmental performance of enterprises, while the impact of enterprises in the central and western regions is more obvious. Compared with the heavy-polluting enterprises, the state’s environmental subsidy effect on the non-serious polluting enterprises is more obvious, and the quality of internal control has the most significant impact on the degree of environmental pollution.

### 5.2. Suggestions

On this basis, the author puts forward four policy recommendations:

Firstly, we should increase the punishment of heavy-polluting enterprises, improve the pollution charge system, and implement different taxes among regions. This paper proposes the current sewage discharge charging system in China, which has a greater impact on environmental performance than environmental subsidy. Therefore, from the actual point of view, in-depth analysis of the difficulties of heavy-pollution enterprises to participate in environmental management, and based on this work out reasonable tax collection standards, strengthen the implementation of environmental protection policies, and give full play to the role of environmental protection tax “Reversed transmission”. When implementing “environmental taxes”, we should bring into the role of public opinion and the media, and encourage heavily polluting enterprises to actively carry out energy conservation and emission reduction and environmental protection technology innovation through exposure and punishment. At the same time, we should strengthen the openness of administrative law enforcement, broaden the channels of supervision and reporting by the masses, strengthen communication with the public and the media, and prevent law-enforcement personnel from violating regulations [98,99,100,101,102].

Second, more support should be given to environmental technology innovation and development, rather than to simply direct investment to enterprises, so as to achieve a “win-win”. By INCREASING the INTENSITY of technological innovation, strengthening the audit of environmental protection technology, protecting the patent of environmental technology, and establishing a perfect evaluation mechanism, enterprises can increase economic benefits through technological transformation [103,104,105,106].

Third, The State Environmental Protection Bureau, the Ministry of Finance and other departments should fully consider the heterogeneity of enterprises with a large environmental impact when providing subsidies, and subsidies should be given to companies with better resource bases. Environmental supervision should be strengthened to overcome inertia. At the same time, strict supervision should be carried out on the use of environmental protection funds to prevent some enterprises from using the “rent-seeking” way to cheat environmental protection funds. For companies with a poor resource base, it is important to expand access to finance, strengthen credit support, reduce credit discrimination and provide financing for environmental technology innovation [107,108,109,110].

Fourth, the property rights of state-owned enterprises determine their role in environmental governance. Therefore, for state-owned heavy-polluting enterprises, more policies and measures should be adopted, stricter pollution emission standards should be formulated, and supervision of their manufacturing processes should be strengthened to reduce pollutant emissions and improve environmental protection for the managers of state-owned enterprises. On the other hand, private enterprises are sensitive to economic pressure because they have undertaken a large amount of investment in environmental protection, and it is difficult for them to get government subsidies, which leads to their lack of investment in environmental protection and technological innovation. Therefore, in practice, one should consider breaking the ownership differentiation, avoiding misappropriate state-owned enterprises environmental subsidies waste phenomenon such as shortage of funds and private enterprise environmental protection investment, encouraging private enterprises to actively undertake social responsibility, with the practical action of environmental protection to state subsidies, winning more environmental subsidies, and improving the environmental competitiveness of enterprises.

## Figures and Tables

**Table 1:** Annual, regional and Industry distribution.

Year	Sample Size	In the East	In the Middle	In the West	Production	Agriculture and Forestry, Animal Husbandry, Fishery	Mining	The Manufacturing and Supply Industry of Electricity, Heat, Gas and Water	Other Industries
2010	317	190	63	63	232	8	12	28	35
2011	348	209	67	72	262	7	14	26	38
2012	341	208	60	73	249	9	11	32	38
2013	360	216	66	77	267	8	11	34	38
2014	364	228	61	74	270	5	10	39	38
2015	416	249	74	91	310	11	12	36	45.9
2016	428	259	76	92	311	14	13	37	52.02
2017	519	323	88	107	378	8	18	45	68.34
2018	564	348	108	107	419	8	14	41	80.58
2019	501	321	85	84	370	8	15	29	78.54
Sum	4161	2555	761	844	3071	87	132	353	516.12

**Table 2:** Variable definition table.

The Variable Type	Variable Name	The Name of the	Definition and Description
Interpretable variable	Corporate Environmental Performance	CEP	Ln (Corporate environmental performance +1)
Interpretation of variables	Government Environmental subsidy	ESUB	Ln (Government environmental subsidy +1)
Adjust the variable	Internal control quality	IC	The internal control index of the enterprise
	Years of operation	AGE	Observe Year—Build year +1
	Growth in operating income	GROW	(year—last year’s)/Last year’s profit
Control variables	Corporate debt level	LEV	Total debt/total assets
	Level of cash reserves	CASH	Net operating cash flow/total assets
	Total return on assets	ROA	Net profit after tax/total assets
	Business interests	CE	operating/Operating expenses
	The ratio of independent directors	IDR	The independent director/The total number of directors
	Year	YEAR	In 2010, nine dummy variables were set
	Industry	IND	According to the China Securities Regulatory Commission (2012 edition) “Guidance on Industry Classification of Listed Enterprises”, there are 13 industry dummy variables

**Table 3:** Correlation Coefficient.

(1)							
Variable	CEP	CEP	IC	LEV	ROA	GROW	AGE
CEP	1.224						
CEP	0.176 ***	1.224					
IC	0.158 ***	0.076 ***	1.224				
LEV	0.292 ***	0.172 ***	0.024	1.224			
ROA	−0.002	−0.09 ***	0.181 ***	−0.554 ***	1.224		
GROW	0.016	−0.0036	0.099 ***	0.022	0.247 ***	1.224	
AGE	0.087 ***	0.053 ***	0.087 ***	0.137 ***	−0.055 ***	−0.057 ***	1.02
CASH	0.059 ***	0.052 ***	0.076 ***	−0.168 ***	0.391 ***	0.022	0.098 ***
CE	−0.142 ***	−0.124 ***	0.054 ***	−0.424 ***	0.501 ***	0.079 ***	−0.014 ***
DUAL	−0.043 ***	−0.103 ***	−0.070 ***	−0.177 ***	0.086 ***	0.010	−0.076 **
IDR	−0.033 *	−0.057 ***	0.019	−0.015	0.027	−0.002	0.03
MP	−0.27 ***	−0.186 ***	−0.172 ***	−0.492 ***	0.070 ***	−0.034 *	−0.136 **
MCH	0.072 **	0.038 **	0.008	0.130 ***	−0.142 ***	0.050 ***	0.074 **
(2)							
Variable	CASH	CE	DUAL	IDR	MP	MCH	
CASH	1.224						
CE	0.244 ***	1.224					
DUAL	0.018	0.045 **	1.224				
IDR	0.006	0.015	0.099 ***	1.224			
MP	−0.098 ***	0.357 ***	0.171 ***	−0.019	1.224		
MCH		−0.073 ***	−0.097 ***	0.037 **	−0.067 ***	1.224	

Note: *** has significance at 1%, ** has significance at 5%, * has significance at 10%.

**Table 4:** Regression analysis of East and mid-west Enterprise Clusters.

Variable	CEP (Model (1))		CEP (Model (2))		CEP (Model (3))	
	The Eastern Region	Central and Western Regions	The Eastern Region	Central and Western Regions	The Eastern Region	Central and Western Regions
ESUB	0.021 **	0.031 **			0.008	0.012
	(3.20)	(4.14)			(1.12)	(1.38)
IC			0.094 ***	0.126 ***	0.081 ***	0.099 ***
			(3.21)	(4.03)	(2.75)	(3.15)
ESUB*IC					0.004 *	0.010 ***
					(2.53)	(3.58)
controls	YES	YES	YES	YES	YES	YES
constant	14.42 ***	13.54 ***	13.96 ***	12.95 ***	13.88 ***	13.07 ***
	(23.94)	(23.38)	(22.12)	(21.09)	(22.01)	(21.31)
Year/Industry	YES	YES	YES	YES	YES	YES
N	2555	1606	2555	1606	2555	1606
Adj.R^2^	0.134	0.217	0.134	0.213	0.139	0.250
F	11.27	13.61	11.15	13.63	11.09	13.85

Note: *** has significance at 1%, ** has significance at 5%, * has significance at 10%.

**Table 5:** Industry categorical regression analysis of heavy and non-heavy pollution.

Variable	CEP (Model (1))		CEP (Model (2))		CEP (Model (3))	
	Heavy Pollution	Non-Heavy Pollution	Heavy Pollution	Non-Heavy Pollution	Heavy Pollution	Non-Heavy Pollution
ESUB	0.024 **	0.0552 ***	0	0	0.0036 *	0.0504 ***
	(3.58)	(4.91)			(0.45)	(3.86)
IC			0.151 ***	0.046 **	0.134 ***	0.041
			(6.39)	(1.03)	(5.60)	(0.92)
ESUB* IC					0.008*	0.002
					(3.99)	(0.68)
controls	YES	YES	YES	YES	YES	YES
constant	19.90 ***	14.59 ***	18.93 ***	14.49 ***	18.96 ***	14.24 ***
	(51.80)	(20.73)	(46.12)	(18.62)	(46.16)	(19.08)
Year/Industry	YES	YES	YES	YES	YES	YES
N	2714	1447	2714	1447	2714	1447
Adj.R^2^	0.176	0.082	0.184	0.068	0.191	0.082
F	28.15	7.03	29.52	5.85	28.21	6.43

Note: *** has significance at 1%, ** has significance at 5%, * has significance at 10%.

**Table 6:** Group regression results of state-owned enterprises and non-state-owned enterprises.

Variable	CEP (Model (1))		CEP (Model (2))		CEP (Model (3))	
	State-Owned Enterprises	Non-State-Owned Enterprises	State-Owned Enterprises	Non-State-Owned Enterprises	State-Owned Enterprises	Non-State-Owned Enterprises
ESUB	0.037 ***	0.0034 ***			0.023	0.016
	(3.60)	(3.55)			(1.69)	(0.67)
IC			0.143 ***	0.089 **	0.128 ***	0.068 *
			(4.38)	(2.66)	(3.86)	(1.96)
ESUB* IC					0.015 ***	0.023 ***
					(2.60)	(3.44)
controls	YES	YES	YES	YES	YES	YES
constant	14.37 ***	11.27 ***	13.69 ***	13.67 ***	13.61 ***	13.61 ***
	(25.72)	(5.41)	(23.09)	(20.20)	(22.91)	(20.18)
Year/Industry	YES	YES	YES	YES	YES	YES
N	2038	2064	2038	2064	2038	2064
Adj.R2	0.191	0.135	0.194	0.132	0.215	0.141
F	13.35	9.30	13.58	9.01	13.39	9.20

Note: *** is significant at 1%, ** is significant at 5%, and * is significant at 10%.

## Data Availability

The data are not publicly available due to privacy restrictions.
